# Doctor and Patient

**Published:** 1921-01-08

**Authors:** 


### Doctor and Patient.

### WHY AN OLD DIFFICULTY IS REVIVED.

The question of the preservation of the secrecy of a patient's confidence entrusted to his doctor is one that recurs in an acute form from time to time, usually in some individual case of public interest in which an attempt is made either in the Courts or elsewhere to induce a medical man to betray that confidence. It is a problem in its general practice and application not so difficult as it seems, for its solution depends in the main on the honour and common-sense of the general practitioner or the specialist, and instances in which the patient's confidence is improperly abused are so rare that they are altogether negligible. Probably in no country in the world are the relations between doctor and patient so excellent as they are in Great Britain, because here the doctor is absolutely trusted, and he rarely fails to justify that trust.

The situation which has now revived the problem in a practical form is one brought about, as usual, not by anything the medical profession has done. It is due to the entirely well-intentioned action of the Ministry of Health in putting forward a system of medical records by which Insurance doctors will be under the compulsion of disclosing in writing the medical details concerning their patients and submitting them for statistical and other purposes to official examination. One can readily see that a system of this kind may develop into a valuable instrument for the treatment of disease; but it seems *primâ facie* to strike such a blow at the tradition of medical secrecy that it has raised a certain amount of criticism in all parts of the country, from medical men as well as patients. So much so that the Ministry have become alarmed, and have deemed it necessary to issue an official statement with the object of reassuring patients. They emphasise in their statement the importance to medical science of a continuous record of a patient, and declare that the new system precludes the possibility, without improper tampering with sealed envelopes, of the prejudicial disclosure of a patient's illnesses.

"It is not to be believed," they say, "that responsible officials will be so carried away by impertinent curiosity as to be guilty of an impropriety, particularly as the confidential nature of the records has been emphasised in the instructions to Insurance Committees." They add that the system impugned has been carefully devised by an expert and mainly medical committees with the express object of meeting the desire of the profession for records that will be of real value in the treatment of disease, and in the advancement of medical knowledge, and at the same time of preserving, in a way, that the patients themselves are likely to desire that secrecy as to medical particulars on which the medical profession itself has rightly always laid such stress.

These are in themselves reassuring words, but the danger is lest the general body of insurance patients should cling to the suspicion that it would be imprudent on their own account to make a full revelation to their doctors, and so be insensibly led to keep back important details that might have a vital bearing on their case. In other words the tendency would be to break down the general confidence which now generally prevails in the sanctity of patients' secrets. It is, therefore, of the first importance that the safeguards indicated in the Health Ministry's memorandum should be clearly set forth in every detail, and that some general expression of opinion as to their value should be elicited from the great body of panel doctors who are intimately concerned in the matter. The record system will of course entail an enormous amount of additional labour on the doctors, and that, no doubt, will have to be taken into account. But of much greater consequence is the strict preservation of the confidence of the patients at large.

